# The correlation between supermarket size and national obesity prevalence

**DOI:** 10.1186/s40608-014-0027-z

**Published:** 2014-12-17

**Authors:** Adrian J Cameron, Wilma E Waterlander, Chalida M Svastisalee

**Affiliations:** World Health Organization (WHO) Collaborating Centre for Obesity Prevention, Deakin University, Melbourne Burwood Campus, 221 Burwood Highway, Burwood Victoria, 3125 Australia; National Institute for Health Innovation, School of Population Health, The University of Auckland, Auckland, New Zealand; Metropolitan University College, Copenhagen, Denmark

**Keywords:** Supermarket, Store size, Food environment

## Abstract

**Background:**

Supermarkets provide healthy and affordable food options while simultaneously heavily promoting energy-dense, nutrient-poor foods and drinks. Store size may impact body weight via multiple mechanisms. Large stores encourage purchasing of more food in a single visit, and in larger packages. In addition they provide greater product choice (usually at lower prices) and allow greater exposure to foods of all types. These characteristics may promote purchasing and consumption. Our objective was to assess the relationship between supermarket size and obesity, which has rarely been assessed.

**Results:**

Data on supermarket size (measured as total aisle length in metres) was from 170 stores in eight developed countries with Western-style diets. Data for national obesity prevalence was obtained from the UK National Obesity Observatory. We found a strong correlation between average store size and national obesity prevalence (r = 0.96).

**Conclusions:**

Explanations for the association between store size and national obesity prevalence may include larger and less frequent shopping trips and greater choice and exposure to foods in countries with larger stores. Large supermarkets may represent a food system that focuses on quantity ahead of quality and therefore may be an important and novel environmental indicator of a pattern of behaviour that encourages obesity.

## Background

Supermarkets are responsible for the provision of a large percentage of the food consumed in most Western countries and typically provide a variety of healthy and affordable food options. At the same time, supermarkets also heavily promote energy-dense, nutrient poor snack foods and soft drinks [1,2]. The consumption of such products is considered a major driver of the global epidemic of obesity [3].

Factors including the density of supermarkets, their distance from home and characteristics of the within-store environment such as availability, price and variety of food products have all been investigated as determinants of purchasing patterns, diet and/or body weight, with mixed findings in different countries. The impact of supermarket size on these outcomes, however, has rarely been considered. A theoretical basis for an association between store size and body weight exists, with individuals being potentially more likely to purchase (and store at home) more food and larger packages of food when shopping in larger stores, and larger stores providing greater product choice, greater exposure to foods of all types and potentially lower prices, each of which may promote purchasing and consumption. In a recent French study among 7,131 shoppers at 1097 supermarkets, Chaix et al. found that shoppers at large hypermarkets had greater body mass index and waist circumference than those at “citymarkets” (smaller stores found in city centres) [4], while a Canadian study found no association between store size and body weight, although that study was among shoppers from only five supermarkets [5]. Here, we use data on supermarket size from a previous eight-country supermarket study to investigate the macro-environmental association between average supermarket size and national obesity prevalence.

## Methods

Data on store size was taken from our recent publication on the availability of snack foods in international supermarkets [2]. In that study a total of 170 supermarkets were audited between 2010 and 2012 across selected cities in eight developed countries with Western style diets (Australia (n = 35), Canada (28), Denmark (18), England (8), Netherlands (20), New Zealand (10), Sweden (19), United States of America (USA) (32)). Supermarkets were sampled equally from neighbourhoods within the least and most socioeconomically disadvantaged areas in Australia, Denmark, the Netherlands and Canada (Montreal only). While all auditors were instructed to try to obtain a sample that was representative of their local area (Geographically, and in terms of the types/chains of supermarkets present), no further explicit sampling criteria were followed. The precise supermarket retailers included in the audits were a reflection of the range of chains present in that location. In some countries this meant only a small number of chains were sampled whereas in other areas with greater diversity in the supermarket sector, multiple chains were sampled. Auditors were self-selected researchers interested in the measurement of the within-store supermarket food environment and sampled from their cities of residence or other region convenient to them. Areas sampled should therefore be considered a convenience sample.

### Store size, shelf space and obesity

As reported previously [2], total store size was calculated as total length of all aisles in the supermarket measured using a measuring wheel, measuring tape, or calibrated paces (in stores from the Bethesda/Washington DC area only). Total aisle length dedicated to each of four snack food and beverage groups (potato chips, chocolate, confectionery and soft drinks) as well as fruit/vegetables was also measured. Data for national obesity prevalence (defined as body mass index (BMI) ≥30 kg/m2) was obtained from the UK National Obesity Observatory with measured data from four countries (Australia, England, New Zealand, USA) and self-reported data from other countries [6]. In 2013, Pearson’s correlation coefficient (r) between average store size and national obesity prevalence was calculated. We also calculated r for the association between obesity prevalence and the supermarket shelf space devoted to chocolate, confectionery, chips, soft drink and fruits/vegetables, both unadjusted and when adjusted for store size [2].

## Results

**Figure 1:**
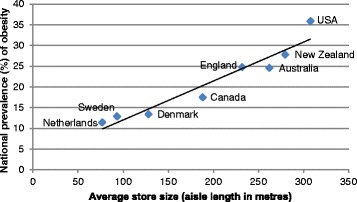
**National obesity prevalence (BMI ≥ 30 kg/m** ^**2**^ **) and average supermarket store size (aisle length in metres) in eight countries.**

A strong positive correlation between average store size and national obesity prevalence was observed (r = 0.96) (Figure 1). This relationship was unchanged using obesity prevalence data sourced from the CIA world factbook (r = 0.95) [7], or the age-standardized prevalence of either overweight or obesity from the World Health Organization database (both r = 0.96) [8]. Raw shelf space allocated to chips (crisps) and soft drink (r = 0.77 and r = 0.78 respectively), was more strongly related to the prevalence of obesity than fruits/vegetables (r = 0.59), chocolate or confectionery (r = 0.19 and r = 0.04 respectively). Shelf space of each of these products after adjustment for total store size (i.e. reflecting the proportion of the store devoted to these products) was not strongly associated with national obesity prevalence (all correlations r < 0.3).

## Discussion

Our demonstration of an extremely strong positive association between the size of supermarkets and national obesity rates suggests that either the large supermarkets themselves, or an aspect of the urban environment or food/shopping culture that encourages such stores, is particularly obesogenic. Our study is of course ecological in nature meaning that a causal relationship cannot be assumed. Nevertheless, it is interesting to speculate as to why this linear relationship was observed. Typically, large supermarkets encourage the purchasing of large amounts of food and less regular shopping trips, usually involving a car. Such shopping behaviour may lead to the purchasing of larger amounts of food of all sorts for storage at home and the purchasing of food in larger sizes and quantities. Large supermarkets can afford to devote far greater shelf space to larger and therefore more economical packets of chips and soft drinks for example, with our finding of a correlation between shelf space of chips and soft drinks (but not chocolate and confectionery) with obesity prevalence supporting this contention. With less frequent shopping trips, larger supermarkets may also encourage greater purchasing of packaged, ultra-processed foods [9] and reduced purchasing of food with shorter shelf-life such as fresh fruit, vegetables and dairy.

From our data, it is clear that the lowest store size and obesity rates were from the three northern European countries sampled. These countries have high urban population density combined with infrastructure that encourages shopping trips by foot, by bicycle or by public transport rather than by car [10]. As a result, shopping trips in these countries may be more frequent, involve active transport and the carrying of groceries, and be more likely to include the purchasing of fresh, perishable foods. As such, large supermarkets may be a useful marker for a cultural and social pattern of shopping and consumption that promotes obesity [11].

The included countries have a Western-style diet and a similar level of economic development in common, with each having GDP per capita among the top 26 countries in the world [12]. Despite the possibility that percentage of GDP spent on food differs between the countries, because of the overall economic similarity it is therefore more likely that the three-fold differences in obesity prevalence are due to differences in environment or culture. Limitations of this study, apart from its ecological nature, include the inability to distinguish between shelf-space allocated to food and non-food items. It would be of interest to confirm the findings seen here in a larger sample of supermarkets from a greater number of countries, and using individual-level shopping and body weight data. The economic, social and other drivers that encourage construction of large supermarkets would also be a research topic of interest. A further limitation of the study is that we cannot be sure that the supermarkets audited are truly representative of all supermarkets in each country. This is particularly the case for England (n = 8) where sample sizes were low both in total and per chain. For some other countries with considerable diversity in the supermarket retail sector (Canada, US) the average store size may vary in different regions. Having said this, sampling in both of these countries was from multiple cities/regions (two in Canada, three in the US). For countries where a larger number of stores from individual chains were audited (e.g. Australia, the Netherlands, Sweden), the results are likely to be representative of the store size of that chain (in each case the leading retailers were selected). It is worth noting that auditors were asked to obtain a sample that was representative of the major supermarkets in their city/region. Given the potential limitations, the results on store size should be considered instructive but not necessarily definitive. Further work to more accurately measure supermarket store size in different countries would be extremely useful to confirm our findings.

## Conclusions

In conclusion, these results suggest that national obesity prevalence in the countries surveyed is correlated with supermarket store size. Large supermarkets may represent a food system that focuses on quantity ahead of quality and therefore may be an important and novel environmental indicator of a pattern of behaviour that encourages obesity.
